# Novel biostimulant bacterial exopolysaccharides production via solid-state fermentation as a valorisation strategy for agri-food waste

**DOI:** 10.1007/s11356-024-34435-y

**Published:** 2024-07-24

**Authors:** Enric Garcia-Muchart, Oscar Martínez-Avila, Laura Mejias, Eline Gilles, Chloé Bluteau, Lucie Lavergne, Sergio Ponsá

**Affiliations:** BETA Tech Centre (TECNIO Network), University of Vic-Central University of Catalonia, Ctra. de Roda 70, 08500 Vic, Spain

**Keywords:** Circular economy, Waste valorisation, Agri-food by-products, Solid-state fermentation, *Burkholderia cepacia*, Biostimulants, Exopolysaccharides

## Abstract

Bacterial exopolysaccharides (EPS) are extracellular polymer-based substances recently defined as potential plant biostimulants, as they can increase nutrient uptake, water retention, and resistance to abiotic stress. As sugar-based substances, the bacteria producing them need to grow in a sugar-rich substrate. Hence, some agri-food by-products could be used as suitable carbon sources for EPS production as a cost-effective and more sustainable alternative to conventional substrates. Thus, this study aimed to produce EPS from specific bacterial strains through solid-state fermentation (SSF) using agri-food waste as a low-cost substrate. Six residues and five bacterial strains were tested in a lab-scale SSF system. From the assessed substrate-strain combinations, *Burkholderia cepacia* with ginger juice waste (GJW) resulted in the most promising considering several process parameters (EPS production, cumulative oxygen consumption, biomass growth, reducing sugars consumption). Also, dynamic monitoring of the system allowed for establishing 5 days as a suitable fermentation time. Then, using response surface methodology (Box-Behnken design), the process was optimised based on airflow rate (AF), inoculum size (IS), and micronutrient concentration (MN). In this stage, the best conditions found were at 0.049 (± 0.014) L h^−1^ per gram of dry matter (DM) for AF, 8.4 (± 0.9) E + 09 CFU g^−1^ DM for IS, and 0.07 (± 0.01) mL g^−1^ DM for MN, reaching up to 71.1 (± 3.2) mg crude EPS g^−1^ DM. Results show the potential of this approach to provide a new perspective on the value chain for the agri-food industry by introducing it to a circular economy framework.

## Introduction

A generalised concern about the need to supply food to an exponentially growing population has emerged as a primary global objective in recent years. Consequently, efficient land use, improved yields, higher-quality production, and reduced environmental and social impacts of agriculture have become the primary focus of scientists and producers (Chojnacka 2015). For example, conventional chemical fertilisers are not environmentally sustainable (Chen 2006), whereas alternatively, organic fertilisers from diverse organic sources and side streams are increasingly being used to reduce these inconveniences. However, due to their chemical bonding form, plants cannot easily absorb some nutrients from organic fertilisers (Halpern et al. 2015).

In this context, a group of substances able to promote plant growth called biostimulants have emerged as key subjects to enhance nutrient uptake efficiency, soil structure stability, and horticulture and agricultural crop quality (du Jardin 2012; Rouphael and Colla 2020). Recently, in the EU Regulation 2019/1009, plant biostimulants were described according to some functional positive effects on plants or plants rhizosphere as “products which improve nutrient use efficiency, tolerance to abiotic stress, quality traits or availability of confined nutrients in the soil or rhizosphere” (European Commission 2019). Considering this stimulation impact, plant biostimulants are expected to play an essential role in sustainable agriculture and organic farming, reducing the difference with conventional agriculture yields (De Pascale et al. 2017). Regardless of their function-based definition, biostimulants are categorised into six main groups, including microorganisms and substances: humic and fluvic acids, protein hydrolysates and other N-containing compounds, seaweed extracts and botanicals, chitosan and other biopolymers, inorganic substances, and beneficial fungi and bacteria (du Jardin 2015).

Polysaccharides, a novel classified substance as a potential plant biostimulant biopolymer, have interesting properties in biosorption, biodegradability, and water retention (More et al. 2014). These are commonly obtained by extraction from biofilm matrixes formed by algae and microalgae species (Xiao and Zheng 2016). However, the low yield reached with algae is one of the significant limitations of their industrial production (Liu et al. 2016). Thus, using bacterial exopolysaccharides (EPS) is a promising alternative for industrially obtaining similar polysaccharides. EPS have been previously studied in several applications in the food and healthcare industry as gelling, emulsifier or thickener agents (Rehm 2010). Moreover, EPS produced by *Pseudomonas* and *Burkholderia* strains have been found to play a crucial role in the persistent antibiotic tolerance of their producing bacteria (Gunardi et al. 2021; Liang et al. 2023). EPS are bacterial extracellular polymers produced in response to environmentally stressful conditions regarding the culture’s pH, temperature, oxygen and micronutrient availability, and C/N ratio (Liu et al. 2004). The interest of EPS as biostimulants has emerged due to their efficiency in conferring resistance to abiotic stress, increasing the nutrient uptake and water retention capacity of plants (Bhagat et al. 2021), as well as improving the soil particle aggregation and quality (Costa et al. 2018). Structurally, EPS are complex biomolecules formed by polysaccharides, sugars, proteins, nucleic acids, lipids, pyruvates, and humic substances. Their main structure contains repeated sequences of one or more monosaccharide units, forming two groups in which EPS are classified: homopolysaccharides and heteropolysaccharides (Donot et al. 2012). Considering such a constitution, the multiple-producer bacteria need to grow in a sugar-rich substrate, which can be an efficient carbon source (Whitfield and Valvano 1993; Cerning et al. 1994). Additionally, for the EPS biosynthesis pathway, bacteria need access to simple sugars (primarily monosaccharides) and phosphate molecules for phosphorylation (Kumar et al. 2007; Freitas et al. 2011). Even with the high efficiency and yield, bacterial EPS production faces the challenge of high economic implementation costs, specifically coming from the raw materials, typically sucrose and glucose, or less frequently, xylose, galactose, and lactose (Roca et al. 2015). Besides, using single or mixed bacterial cultures is another relevant aspect influencing EPS productivity and operational costs (More et al. 2014). Although using mixed cultures has proven to be more efficient and economical in several fermentation applications, evaluating the performance of single strains is still a widespread approach to determine their behaviour and yield. After such a step, further investigations usually explore the mixed cultivation of the selected cultures with other microorganisms as a potential parameter to enhance EPS productivity (Ng et al. 2024).

An attractive option to make EPS production more economically and environmentally sustainable could be through alternative substrates such as agri-food wastes acting as a suitable sugar source that, at the same time, help to reduce the production cost (Joulak et al. 2022). Additionally, using agri-food residues as substrates provides a new perspective on the value chain for the agri-food industry by introducing it to a circular economy framework. It is known that agri-food industries generate a massive amount of solid waste during processing (147.2 million tons worldwide), and such waste, if not properly managed, can induce environmental pollution (Sadh et al. 2018). However, due to their richness in organic and inorganic matter, waste and by-products can be used as alternative carbon and nitrogen sources to produce valuable and marketable bioproducts, such as biopolymers. Thus, the bioconversion of waste and by-product streams is a trending topic addressed from different perspectives. For instance, bioflocculants production from sludge and livestock wastewater by aerobic bacterial growth (Peng et al. 2014), poly-β-hydroxybutyrate production from corn waste and rice straw (Sayyed et al. 2021), polyhydroxyalkanoates from activated sludge (Zhang et al. 2023), or cellulose nanocrystals from red banana peduncle waste through chemical extraction (Nagarajan et al. 2022). Moreover, if these bioproducts can be reintroduced in the same value chain as new bio-based packaging, biostimulant products, or food additives (emulsifiers or gelling agents), two agri-food industry challenges would be managed simultaneously.

On the other hand, if low-cost substrates are coupled with technologies capable of improving bioconversion performance, a more effective and sustainable circular economy approach can be implemented. Therefore, solid-state fermentation (SSF) appears as an alternative approach, previously successful as a valorisation strategy for transforming waste streams into diverse value-added bioproducts of industrial interest such as biopesticides (Mejias et al. 2020), bioplastics (Martínez-Avila et al. 2021), enzymes (Mejias et al. 2018; Llimós et al. 2022), and aroma compounds (Martínez et al. 2017), among others. Bacterial EPS have also been produced by SSF in some proof-of-concept studies using fruit-based and grain-based substrates as solid media, reaching a maximum yield of 61.4 mg EPS per g of dry matter (DM) (Stredansky et al. 1999; Guérin et al. 2023).

SSF is carried out in the absence (or near absence) of free water, and it is characterised by requiring lower water and energy consumption, high productivity, and reduced waste generation (Bhargav et al. 2008; Soccol et al. 2017); its main competitive advantages compared to the conventional submerged fermentation. On the other hand, since it is a solid-phase system, SSF could lack proper homogeneity and low mass and energy transfer, affecting process yield at higher scales (Cerda et al. 2019). However, these challenges can be faced, and then favourable processes can successfully produce desired products from solid wastes used as substrates for the fermentation of known microorganisms (Couto and Sanromán 2006).

Thus, the main objective of the present study is to produce EPS from specific bacterial strains through SSF using agri-food waste as a substrate. In particular, it was intended to determine, among five different bacteria and six agri-food solid substrates, the best strain-substrate combination for producing EPS through a comprehensive screening and then to optimise the selected combination to maximise EPS production at the lab scale. Additionally, it aims to generate enough valuable information to scale up the process and get closer to an industrial perspective. Therefore, a new value-chain perspective for the agri-food industry is proposed, suggesting that the production of EPS from agri-food wastes be used in the same agricultural sector as biostimulants.

## Materials and methods

### Bacterial strains and inoculum

*Azotobacter beijerinckii* (CECT 9204) and *Alicyclobacillus acidocaldarius* (CECT 4328) were acquired from *Colección Española de Cultivos Tipo* (Valencia, Spain), *Leuconostoc mesenteroides* (DSM 20484) and *Geobacillus thermodenitrificans* (DSM 465) were purchased from the *German Collection of Microorganisms and Cell Cultures* (Braunschweig, Germany), and *Burkholderia cepacia* (CCM 2656) was obtained from the *Czech Collection of Microorganisms* (Brno, Czech Republic). The strains were maintained at − 80 °C in cryovials containing treated beads and a cryopreservative solution (Microbank™). Inoculum preparation consisted of inserting one treated bead into a 100-mL Erlenmeyer flask containing 40 mL of a specific liquid culture medium for each strain. For *A. beijerinckii*, a 0.5 g L^−1^ glucose, 0.5 g L^−1^ starch, 0.5 g L^−1^ yeast extract, 0.5 g L^−1^ proteose peptone, 0.3 g L^−1^ K_2_HPO_4_, and 0.05 g L^−1^ MgSO_4_·7H_2_O medium were used after being adjusted to pH 7.2. For *A. acidocaldarius*, the culture medium was composed of 1 g L^−1^ glucose, 1 g L^−1^ yeast extract, 0.07 g L^−1^ CaCl_2_, 1.3 g L^−1^ (NH_4_)_2_SO_4_, 0.37 g L^−1^ K_2_HPO_4_, 0.25 g L^−1^ MgSO_4_·7H_2_O, and adjusted to pH 4. For *L. mesenteroides*, a 10 g L^−1^ beef extract, 10 g L^−1^ casein peptone, 20 g L^−1^ glucose, 5 g L^−1^ yeast extract, 2 g L^−1^ K_2_HPO_4_, 0.2 g L^−1^ MgSO_4_·7H_2_O, and 0.05 g L^−1^ MnSO_4_·H_2_O medium was done and adjusted to pH 6.5. For *G. thermodenitrificans*, the medium was composed of 3 g L^−1^ beef extract, 5 g L^−1^ peptone, 0.01 g L^−1^ MnSO_4_·H_2_O, and adjusted to pH 7. For *B. cepacia*, LB culture medium (Lysogeny Broth, Panreac) was used at 25 g L^−1^, as indicated by the supplier. Flasks with the cultures were placed in an orbital shaker at 120 rpm in aerobic conditions for 48 h. Growth temperatures were set at 30 °C for *A. beijerinckii*, *L. mesenteroides*, and *B. cepacia*, at 55 °C for *A. alicyclobacillus*, and 60 °C for *G. thermodenitrificans*. Cultures were considered ready once they reached the maximum optical density, measured at 600 nm. All materials and reagents were previously sterilised by autoclaving at 121 °C for 20 min.

### Substrates

*CoBeverage Lab* (Catalonia, Spain) supplied beet juice waste (BJW), mainly composed of beet, carrot, apple, and cucumber solid by-products, and ginger juice waste (GJW), which mainly consisted of ginger, apple, and lemon solid wastes. Pomegranate peels (PP) and pomegranate seeds (PS) were provided by *Moleva* (Catalonia, Spain), a local fruit juice producer. Apple pomace (AP) was collected from green apples used for cider production in *Mooma* (Catalonia, Spain), and *Liquats Vegetals* (Catalonia, Spain) procured vegetable milk waste (VMW), a solid mix of soy, oat, and rice residues generated in the vegetable drinks production. All substrates were stored at − 20 °C to keep their original structure. To prepare VMW for the SSF process, sponge cloth (Spontex®) pieces were added in a 10% (*w/w*) ratio to the residue as bulking agents to increase its porosity, as suggested by Puyuelo et al. (2011). For their processing, each material was autoclaved at 121 °C for 20 min before being inoculated with each strain in the proportions defined for each experiment.

### SSF system setup

SSF experiments were performed in a dynamic respirometric system based on Gómez et al. (2005), which consisted of a previously sterilised (by autoclaving at 121 °C for 20 min) 0.5-L reactors (Erlenmeyer flask) connected to a mass flow controller meter (Bronkhorst High-Tech, Netherlands) which forced air to flow through the solid bed from the bottom to the top of the reactor. Before entering the reactor, the air was humidified in a bottle that contained distilled water to prevent samples from drying during fermentation. The reactors, containing 100 ± 1 g of inoculated material (“Substrates” section), were immersed in a water bath set at the optimal growth temperatures for the used strains. The outgoing airflow was conducted through another Erlenmeyer flask, which was working as a water trap, to an oxygen sensor (O2-A2, Alphasense, UK), allowing a programmed data logger software (Arduino® and LabVIEW based) to collect the oxygen concentration every 5 min. Using this data, a respirometric analysis was conducted as described by Ponsá et al. (2010), which involved calculating the specific oxygen uptake rate (sOUR) and the cumulative oxygen consumption in 4 days (COC_4_).

During the screening experiments, the airflow rate was set at 0.6 L h^−1^ g^−1^ DM, and all the substrates were inoculated with 10% (*v/w*) of each bacterial strain. The experiments were followed up for 5 days and performed in duplicates. The time course of the selected combination substrate-strain was conducted for 8 days (8 replicates) to further detail the process’ dynamics, using a single biological replicate for each sampling day.

### Analytical methods

#### Substrates characterisation

The substrates were characterised by conducting the following analyses according to standard procedures (Leege 1998): dry matter (DM), organic matter (OM), total Kjeldahl nitrogen (TKN), water holding capacity (WHC), bulk density (BD), and pH. Also, the CNH percentage was obtained using the elemental analyser, and cellulose, hemicellulose, and lignin were determined by gravimetric methodology (Möller 2009).

#### Bacterial biomass quantification

Bacterial biomass was determined by a gravimetric method, as detailed by Martínez-Avila et al. (2021). From a vacuum-filtered (0.22 µm) sample, obtained after a double consecutive solid–liquid extraction of 5–10 g of sample with distilled water in a 1:3 (*w:v*) ratio at 165 rpm, 30 °C for 35 min, a pellet of biomass was obtained after centrifuging at 4200 rpm, 4 °C for 15 min. Then, the supernatant was discarded or used for reducing sugars analysis, and the pellet, which was placed in a previously weighted tube, was left in an air oven at 60 °C for at least 24 h to dry. After cooling, the tubes were weighted, and the difference was computed for the biomass or cell dry weight (CDW).

#### Reducing sugars analysis

Reducing sugars of the solid substrates and the fermented material were measured using the supernatant collected after a double solid–liquid extraction of the solid samples using distilled water in a 1:3 (*w:v*) ratio at 165 rpm, 30 °C for 35 min (“Bacterial biomass quantification” section). This supernatant was centrifuged at 4200 rpm for 15 min and was diluted before analysis. The quantification was done following the 3,5-dinitrosalicylic acid (DNS) method as detailed by Miller (1959), and the absorbance was measured at 540 nm. The concentrations of reducing sugars were determined using a calibration curve based on known glucose concentrations, which was used as standard.

#### Exopolysaccharides quantification

A modified ethanol extraction method was followed as it is the most used technique due to its high efficiency (D’Abzac et al. 2010). First, 5–10 g of solid substrate was subjected to a double solid–liquid extraction with distilled water in a 1:3 (*w:v*) ratio at 165 rpm for 35 min. The obtained supernatant was filtrated with a muslin cloth and centrifuged (4200 rpm, 4 °C for 20 min). The resultant supernatant was shaken for 45 min at 60 °C after adding NaCl at 5% to denature the protein content (Huang et al. 2011). Then, after cooling to room temperature, centrifuging, and discarding the pellet, cold pure ethanol was added (2:1 ratio) and kept at 4 °C for 24 h. After this period, EPS precipitation was observed in the organic phase, which was collected and centrifuged at 4200 rpm, 4 °C for 20 min. The obtained pellet was dried at 60 °C for 24 h and weighed as the crude EPS in the sample.

#### Carbohydrates quantification

The total carbohydrate content of the crude EPS was determined following the protocol described by Dubois et al. (1956). 3 ± 1 mg of crude EPS were diluted in 9 mL of distilled water and placed in a shaker at 240 rpm and 30 °C to dissolve the EPS pellet properly. Then, 50 µL phenol (80%) solution and 5 mL of concentrated sulfuric acid were added to 2 mL of sample. After 10 min at room temperature, the samples were incubated for 10 min at 30 °C in a water bath. Next, the absorbance was measured at 485 nm, and the glucose standard was used as the calibration curve to quantify pure EPS carbohydrate content.

### Design of experiments and statistical analysis

A Box-Behnken experimental design was used for the optimisation stage. The evaluated factors were set to 3 levels (lower, middle, and higher value), and a triplicate central point was included with a total of 15 experiments performed in two blocks. These experiments were designed and analysed with Design-Expert (DX) v.13 software (Stat-Ease, Inc). The specifically selected range of airflow rate (AF) was 0.03–0.06 L h^−1^ g^−1^ DM, inoculum size (IS) was between 1.0 E + 08 and 1.0 E + 10 CFU g^−1^ DM, and micronutrients concentration (MN) was 0–0.07 mL g^−1^ DM. Preparation of the micronutrient stock solution was conducted by mixing the selected compounds in 100 mL of distilled water as follows: MgSO_4_·7H_2_O 2.03 mg mL^−1^, MnSO_4_·H_2_O 0.25 mg mL^−1^, ZnCl_2_ at 0.17 mg mL^−1^, H_3_BO_3_ at 0.26 mg mL^−1^, and C_6_H_11_FeNO_7_ at 0.39 mg mL^−1^. Interaction among selected parameters was analysed using response surface experimental design. EPS yield, biomass production, reducing sugars consumption, respirometric parameters (sOUR and COC4), and pH variation were also analysed from those experiments, like in the previous experimental stages.

To assess the statistical variances in the experiments, a one-way ANOVA was conducted with a significance level of *p* < 0.05 using the Tukey test. The experiments were performed in triplicates, and the results were presented as mean values ± standard deviation. Data were analysed using SPSS 29.0.0.0 (241) software (IBM Corp.).

## Results and discussions

### Characterisation of the agri-food residues

The selected substrates are agri-food wastes or leftovers chosen based on their potential for producing bacterial EPS. Hence, these included sugar-rich materials with suitable physical properties for a solid-phase process and a specific capacity to promote stressing conditions fostering EPS production. Furthermore, some of these have been previously used for similar aims in SSF processes, showing attractive characteristics (Bhargav et al. 2008; Yazid et al. 2017; Roukas and Kotzekidou 2020).

Table 1 summarises the initial characterisation made on all substrates. As observed, some residues (VMW and PS) contain more nitrogen than others (GJW, AP, PP, and BJW), and consequently, they have a lower C/N ratio. It has been found that at C/N ratios between 20 and 100, EPS production was more efficient (Ye et al. 2011). Moreover, according to Durmaz and Sanin (2001), a low C/N ratio in the substrates indicates a low carbohydrate content in the bacterial EPS. Also, it can be seen that some materials (AP, GJW, and BJW) are richer than others in reducing sugars content, which is a good indicator for potential EPS production (Joulak et al. 2022). In addition, the WHC of each substrate is detailed on a wet basis, and it can be noticed that some of them (GJW, BJW, AP, and PS) are relatively higher than the others. It is an essential parameter to evaluate the potentiality of the substrate to be inoculated and to perform a SSF process (Martínez-Avila et al. 2022). Regarding fibre content, all the substrates show a similar pattern except for PP. While GJW, BJW, AP, PS, and VMW have a higher amount of cellulose fibre, PP has an appreciably higher concentration of hemicellulose. Different cellulose, hemicellulose, and lignin compositions can represent a distinct potential to biochemically digest the fibres and produce diverse valuable bioproducts (Sadh et al. 2018). Thus, it is expected that the more available and simpler the carbon sources are for EPS production, the better the carbon assimilation and EPS assembly. Finally, concerning pH, all feedstocks are pretty acidic, with values between 3.7 and 5.0. The optimum pH value for EPS production depends mainly on the bacterial strain (More et al. 2014), and according to Wani et al. (2021), the range for the maximum yield is between 4 and 5.5. Taking all these parameters into account, on the one hand, BJW, GJW, and AP are the substrates with better characteristics for EPS production through SSF. On the other hand, VMW and PS could be initially discarded due to their low reducing sugars content and C/N ratio. Additionally, PP showed low WHC, impacting the ability to retain the inoculum in the solid phase, and its reducing sugars levels are not particularly high. Thus, it is not foreseen as a promising substrate for bacterial growth.

**Table 1:** Initial composition of used substrates

	Beet juice waste (BJW)	Ginger juice waste (GJW)	Apple pomace (AP)	Pomegranate peels (PP)	Pomegranate seeds (PS)	Vegetable milk waste (VMW)
Dry matter (%)	15.4 ± 0.7	23.0 ± 1.3	18.1 ± 0.9	27.6 ± 3.8	44.4 ± 3.8	17.9 ± 0.3
Organic matter (% DM)	93.9 ± 0.8	96.2 ± 0.6	97.6 ± 0.4	92.0 ± 2.0	92.8 ± 3.0	93.4 ± 0.3
pH	5.0 ± 0.2	4.3 ± 0.3	3.7 ± 0.0	3.8 ± 0.2	4.3 ± 0.0	4.5 ± 0.0
Reducing sugars (g g^−1^ DM)	0.24 ± 0.03	0.35 ± 0.04	0.43 ± 0.07	0.13 ± 0.01	0.09 ± 0.00	0.06 ± 0.01
TKN (g kg^−1^ DM)	13.3 ± 1.5	6.0 ± 1.8	7.2 ± 2.5	7.5 ± 1.8	26.2 ± 2.8	76.8 ± 3.6
Carbon (%)	40.9 ± 1.4	44.8 ± 0.3	44.3 ± 3.6	45.0 ± 0.1	51.6 ± 0.3	44.9 ± 0.0
Nitrogen (%)	1.2 ± 0.2	0.8 ± 0.0	0.7 ± 0.1	0.4 ± 0.0	2.7 ± 0.1	7.0 ± 0.0
C/N ratio	34.2 ± 7.0	58.9 ± 1.9	67.8 ± 8.9	106.7 ± 2.1	19.2 ± 0.7	9.3 ± 3.2
WHC (mL g^−1^)	1.37 ± 0.16	1.87 ± 0.02	1.12 ± 0.22	0.20 ± 0.01	0.83 ± 0.19	0.37 ± 0.10
Density (kg L^−1^)	1.00 ± 0.08	1.03 ± 0.02	0.90 ± 0.06	1.13 ± 0.06	1.07 ± 0.07	1.06 ± 0.12
Cellulose (%)	16.53	16.53	10.77	5.71	12.10	12.28
Hemicellulose (%)	2.32	2.32	5.99	29.21	2.85	3.43
Lignin (%)	3.96	3.96	4.46	2.26	4.80	7.08

*DM*, dry matter; *TKN*, total Kjeldahl nitrogen; *WHC*, water holding capacity. *n* = 3

### Setting the basis to produce bacterial EPS via SSF

#### Substrates and microorganisms screening for EPS production

Considering all the selected and analysed agri-food by-products, they were combined with the five previously chosen bacterial strains to evaluate their potential to produce EPS through SSF. The strains used were selected according to their capability to generate EPS under different conditions. Among them, there is a representative variety in terms of their optimal temperature and pH growth conditions. It has been extensively studied the capacity of lactic acid bacteria to produce EPS (Behare et al. 2009), specifically the selected strain *Leuconostoc mesenteroides* (Savadogo et al. 2004; Yilmaz et al. 2022). Another mesophilic strain, *Azotobacter beijerinckii*, is known to produce two acidic EPS based on D-galactose and L-rhamnose (Likhosherstov et al. 1991). Also, *Burkholderia cepacia* is a well-studied strain characterised by its EPS production versatility in different growth conditions (Cérantola et al. 2000; Cuzzi et al. 2014). Thermophilic strains such as *Geobacillus thermodenitrificans* have also been described as promising sources of EPS (Nicolaus et al. 2000; Panosyan et al. 2018). Lastly, acidophilic bacteria are also good candidates for producing EPS due to their extreme growth conditions (Kuschmierz et al. 2022).

Table 2 gathers some results after SSF from the most relevant and representative substrate-strain combinations regarding the initial screening of strains-substrates combinations. In a general overview, it was found that three combinations stood out among the other ones in terms of crude EPS yield (*A. acidocaldarius*-AP, *B. cepacia*-BJW, and *B. cepacia*-GJW). Since EPS production occurs during bacterial growth, which usually comes from sugars consumption (Joulak et al. 2022), it could be expected that the higher the biomass growth, the cumulative oxygen consumption (COC), and the reducing sugars exhaustion, the higher the EPS production will be. Results show that, on the one hand, among the 30 strain-substrate tested combinations, the highest biomass production was attained by *A. acidocaldarius* and AP, while on the other hand, the best combinations regarding the reducing sugars consumption and pH variation were *B. cepacia* with both juice wastes (GJW and BJW). Even though, once the crude EPS results were analysed, these showed a similar trend, highlighting the combination of AP with *A. acidocaldarius* as the most productive, followed by *B. cepacia* with GJW and BJW, setting a correlation between biomass growth and EPS production as suggested by Turakhia and Characklis (1989). Concerning the cumulative oxygen consumption in 4 days (COC_4_) values, they can be easily differentiated from the combinations with bacterial growth and those with no respirometric activity. In the cases where more than 100 mg O_2_ g^−1^ DM had been detected, a biomass growth or a pH variation was observed that confirmed bacterial activity in the substrates.

**Table 2:** Summary of the main results for the best substrate-strain combinations from the screening experiment

Substrate	Microorganism	Crude EPS (mg g^−1^ DM)	Biomass (mg × g^−1^ DM)	Reducing sugars consumed (%)	pH variation	COC_4_ (mg O_2_ g^−1^ DM)
AP	*A. acidocaldarius*	88.1 ± 3.5	35.7 ± 7.7	0.0 ± 0.0	1.1 ± 0.2	272.4 ± 63.4
BJW	*B. cepacia*	44.9 ± 8.6	15.1 ± 2.3	71.4 ± 6.8	2.2 ± 0.1	301.6 ± 101
GJW	*B. cepacia*	55.4 ± 6.2	16.6 ± 5.4	82.2 ± 3.7	1.5 ± 0.0	242.5 ± 28.9
AP	*B. cepacia*	0.0 ± 0.0	5.0 ± 2.9	21.1 ± 2.3	0.5 ± 0.4	253.6 ± 111
AP	*G. thermodenitrificans*	0.0 ± 0.0	2.3 ± 0.8	0.0 ± 0.0	0.1 ± 0.0	23.4 ± 3.9

*DM*, dry matter; *COC*_*4*_, cumulative oxygen consumption in 4 days (after lag phase)

Although AP – *A. acidocaldarius* was the most EPS-productive combination and the one with the highest biomass, it resulted in a near-cero reducing sugar consumption. Such an outcome could be the consequence of a different metabolisation route in this microorganism. *A. acidocaldarius* is a thermoacidophile bacteria known for its ability to grow on a wide variety of carbon sources, including mono-, di-, oligo-, and polysaccharides (Beck 2020). Its ability to use more complex carbon forms is due to its production of many glycoside hydrolases, including β-1,4-glucanase (Bai et al. 2010), β-galactosidase (Di Lauro et al. 2008; Strazzulli et al. 2017), or cellulases (Morana et al. 2008). Such capacity to produce glycoside hydrolases might have increased the amount of metabolisable sugars during the bioprocess, converting complex carbon configurations such as cellulose or hemicellulose into available carbon monomeric structures. Consequently, the reducing sugar consumption variable was altered for this specific combination, making it unsuitable for comparisons with other scenarios where other bacteria, such as *B. cepacia*, were used. However, in the AP – *A. acidocaldarius* combination, a rapid moisture content loss, mainly induced by the fermentation temperature (55 °C), apparently caused the compaction on the solid, hindering the airflow through the reactor and a suitable mass and heat transfer. Consequently, the oxygen during the active phase of the fermentation decreased to anoxic levels, inducing a sudden cessation of biomass production considering that *A. acidocaldarius* is an obligate anaerobe. Thus, even though the results at the lab scale show high potential for this combination, the foreseen requirements towards a future scale change were taken in mind to choose the second best alternative (*B. cepacia* with GJW and BJW) to detail the proposed system further.

**Fig. 1:**
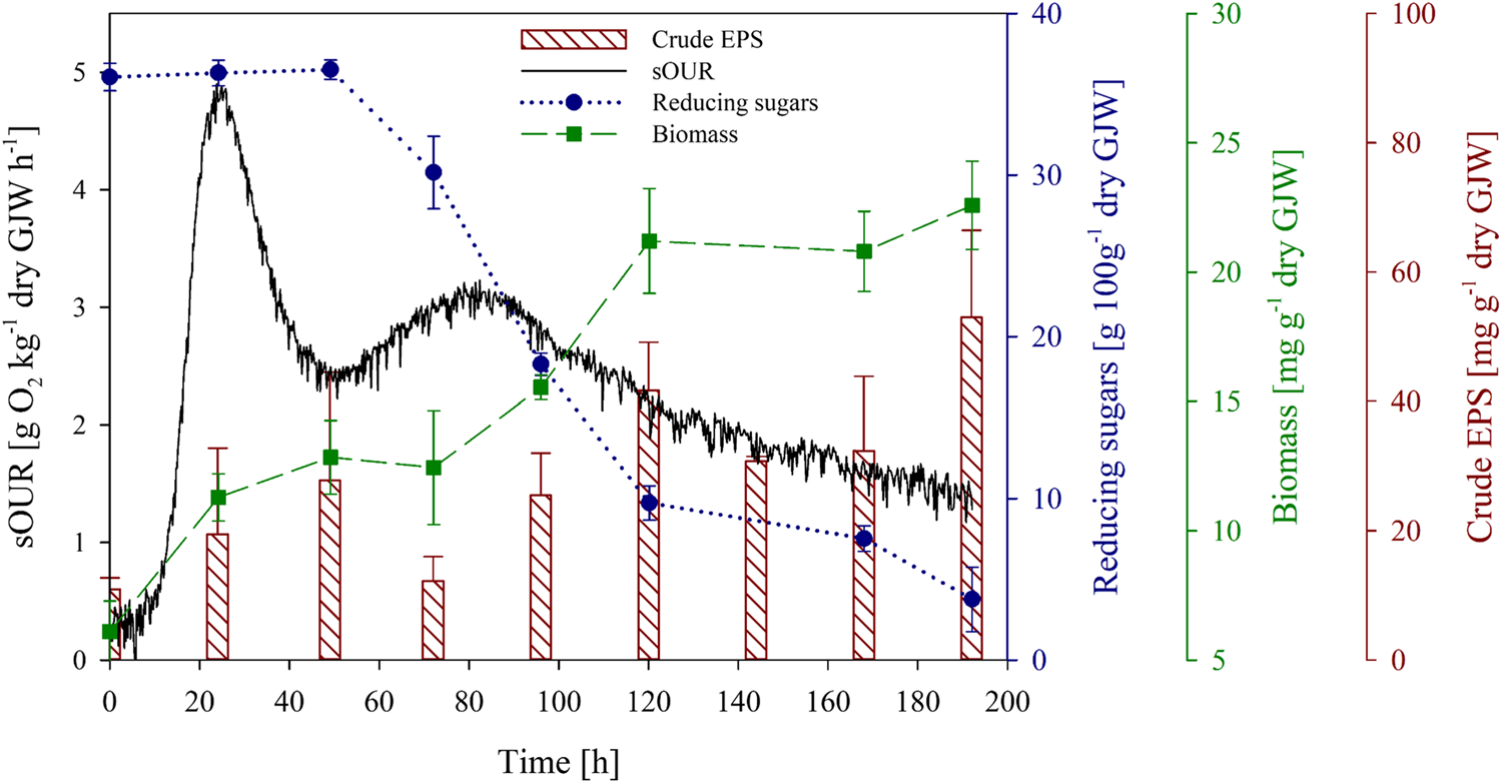
Time course of an 8-day SSF of *B. cepacia* with ginger juice waste (GJW)

Once working with the selected combinations (*B. cepacia* with GJW and BJW), similar results were obtained regarding biomass and EPS production, reducing sugars consumption and specific oxygen uptake rate (sOUR). The combination with a higher EPS production (GJW – *B. cepacia*) was chosen to optimise the time of the process, and a complete time-course study was conducted. In Fig. 1, it can be observed that there is a trend showing a correlation between the measured variables and the EPS production. However, not all the monitored variables vary at the same time because some of them are consequential to others. For example, biomass growth and EPS production increase 24–48 h after oxygen and sugar consumption start. In addition, Fig. 1 shows that the highest EPS production in the given SSF scenario was observed on the fifth and the last day, coinciding with the plateau phases of available sugars consumption and biomass production. Although a drop in EPS yield between 48 and 96 h can be observed, the analysis of the other monitored variables and their similar and correlated trends indicated that it was probably caused by the slightly different biological dynamics of the time-course bioreactor replicates. Additionally, two different sOUR peaks were observed, which were correlated to two increasing phases of biomass production. Based on this complete analysis of the fermentation dynamics, 5 days was selected as the optimum time in which EPS are produced in significant amounts without affecting the productivity of the process. Consequently, the 5-day fermentation of the combination of GJW as a substrate with the bacterial strain *B. cepacia* was the most effective of the evaluated set to produce the biostimulant EPS.

#### Application of a response surface methodology to identify the relevance of some manipulable operational parameters

Based on the previous results, an optimisation experiment employing a Box-Behnken design was conducted using the combination *B. cepacia*-GJW to maximise EPS production while assessing the effects of the specific airflow rate (AF), inoculum load (IS), and micronutrients addition (MN) (Mg, Mn, Zn, Fe, and B). These operational parameters were selected due to their importance in the specific SSF process to produce bacterial EPS. In particular, airflow rate is known to affect solid-based fermentation and composting systems (Mejias et al. 2017; Martínez-Avila et al. 2022); inoculum size has a relevant influence on the starting active bacterial biomass that is injected into the substrates, which can modify the lag phase of bacterial growth during SSF and the exponential growing load of biomass, and consequently their associated bioproducts of interest (Betchem et al. 2023); and the micronutrients concentration which can have an effect directly on the bacterial EPS metabolism (More et al. 2014; Quijada et al. 2022). Other operational parameters, such as pH, temperature, and moisture content, were not considered for the optimisation stage since the aim of the study was to use the potential substrates in the conditions they are usually generated, not significantly modifying pH and moisture content, and because it is expected that the temperature could not be controlled in large-scale SSF systems. Therefore, being unable to exploit the outcomes of such optimisation.

According to the results, the maximum EPS levels achieved were 72.71 mg EPS g^−1^ DM (AF = 0.03 L h^−1^ g^−1^ DM; IS = 5.05 E + 09 CFU g^−1^ DM; MN = 0.00 mL g^−1^ DM) and 70.43 mg EPS g^−1^ DM (AF = 0.045 L h^−1^ g^−1^ DM; IS = 1.00 E + 10 CFU g^−1^ DM; MN = 0.07 mL g^−1^ DM), which were obtained at substantially different operational conditions. It is relevant to highlight that these EPS yields, and all the mentioned in this study, are expressed as crude EPS production values. However, carbohydrate quantification was performed as a direct index of the polysaccharides content of the produced crude EPS. In general, carbohydrate content obtained with the selected combination ranged between 30 and 50%. Nevertheless, the analysis performed in this study was conducted using crude EPS-based results.

Hence, after applying a quadratic model regression with the experimental results of crude EPS production as the response variable, a model in terms of coded factors (A: AF; B: IS; C: MN) (Eq. 1) was obtained. From this model, the obtained coefficients represent the expected change in response per unit change in factor value when the remaining factors are kept constant. There, it can be observed that the parameters with the highest effect on EPS production were inoculum size (*p*-value = 0.0117) and the self-interaction of inoculum size (*p*-value = 0.0233). From these experiments, and to show the actual influence of the optimised parameters on crude EPS production (variable output) with their actual units (AF: L h^−1^ g^−1^ DM; IS: mg EPS g^−1^ DM; MN: mL g^−1^ DM), a polynomial equation in terms of actual factors was obtained (Eq. 2).

1 $$\text{Crude EPS }=47.95+11.37\times \text{B}-4.02\times \text{C}+3.85\times \text{A}\times \text{C}+9.42\times \text{B}\times \text{C}+8.16\times {\text{A}}^{2}-14.30\times {\text{B}}^{2}-4.30\times {\text{C}}^{2}$$

2 $$\text{Crude EPS }=41.33+6.57\times \text{IS}-580.60\times \text{MN}+4750.18\times \text{AF}\times \text{MN}+54.39\times \text{IS}\times \text{MN}+2928.54\times {\text{AF}}^{2}-0.61\times {\text{IS}}^{2}-2956.58\times {\text{MN}}^{2}$$

The significance value for the model was *p*-value = 0.0362, and the lack of fit significance was *p*-value = 0.1211, which means that the model is robust and significant, and it allowed the data to fit appropriately. The observed trend indicates that to achieve maximum production, it is needed to work with high micronutrient concentrations, elevated inoculum load, and nonspecific airflow rates. Particularly, the most desired conditions according to the quadratic model given by the Box-Behnken design were an airflow rate of 0.049 (± 0.014) L h^−1^ g^−1^ DM, an inoculum size of 8.36 (± 0.96) E + 09 CFU g^−1^ DM, and a micronutrients concentration of 0.07 (± 0.001) mL g^−1^ DM, in order to achieve a crude EPS production of 71.1 (± 3.2) mg EPS g^−1^ DM.

**Fig. 2:**
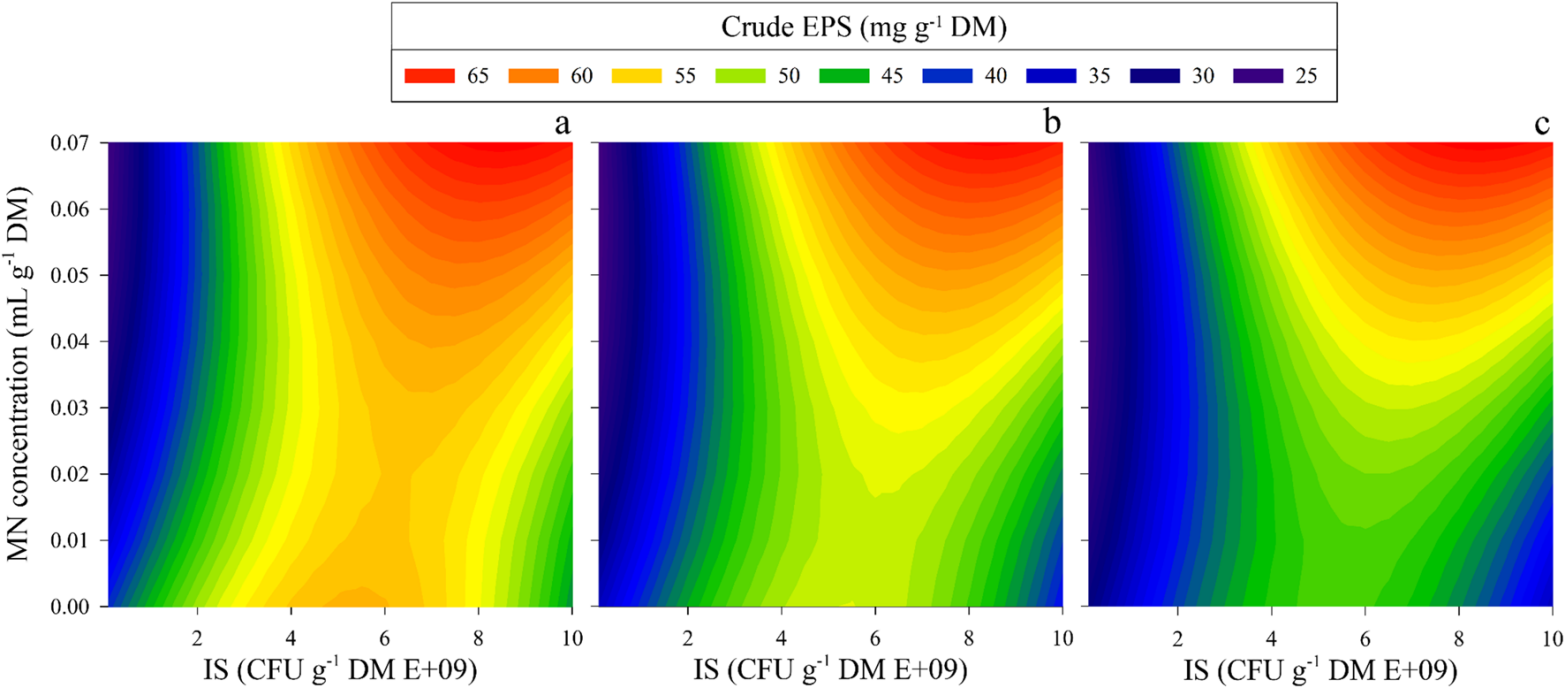
Contour plots for EPS production as a function of inoculum size (IS) and micronutrients concentration (MN). The airflow rate (AF) was set at three levels. **a** AF = 0.03 L h^−1^ g^−1^ DM; **b** AF = 0.045 L h^−1^ g^−1^ DM; **c** AF = 0.06 L h^−1^ g^−1^ DM

**Fig. 3:**
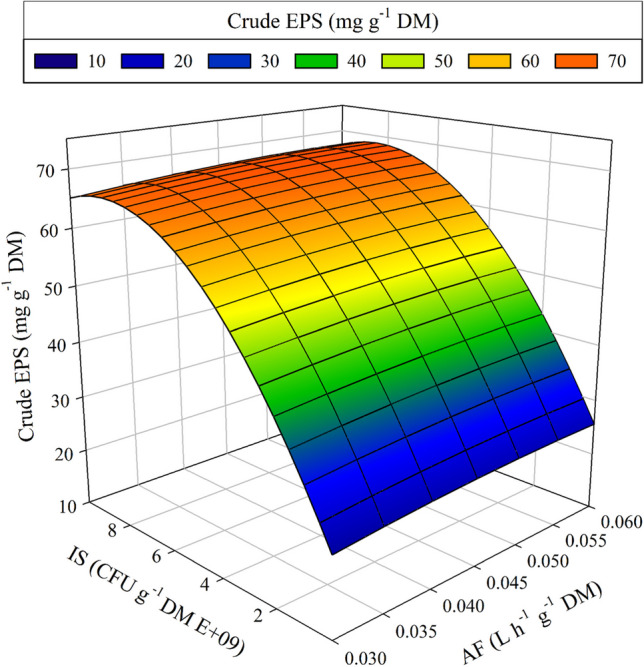
Response surface for EPS production as a function of the airflow rate (AF) and inoculum size (IS)

**Fig. 4:**
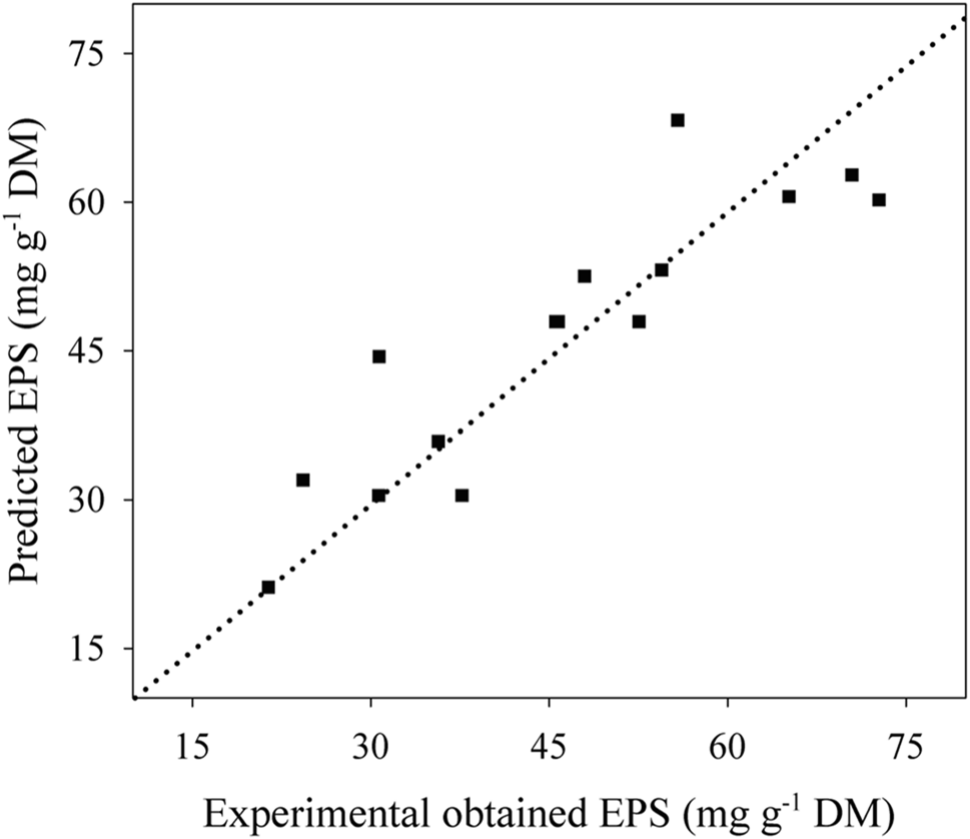
Predicted vs. experimental values of crude EPS production in the optimisation stage. *y* = 0.983*x*; *R*.^2^ = 0.979

A complementary vision of these results can be obtained from the contour plots in Fig. 2. As seen, EPS production is influenced by IS and MN independently of the AF levels. It can be observed that the contour trend is maintained in the three graphs, indicating a non-significance of the AF parameter. Also, it can be noticed that the maximum EPS production is focused on the superior points of the MN axis and the highest values of the IS axis. Therefore, it was confirmed that the optimal MN value was around its maximum (0.07 mL g^−1^ DM), and the most desired conditions of inoculum size were around 7.00 E + 09 and 1.00 E + 10 CFU g^−1^ DM. Thus, to thoroughly understand the EPS production behaviour, a response surface was built in Fig. 3, showing the EPS yield depending on the AF and the IS parameters, setting the MN at the previously observed more productive value (MN = 0.07 mL g^−1^ DM). From this figure, it can be deduced that the tested interval of AF does not significantly affect the EPS yield because even with the minimum AF supply, it maintains enough oxygen to ensure the metabolic activity of the aerobic process. Among the runs conducted, oxygen levels did not reach values below 7%. Sufficient oxygen at these levels indicates that aerobic conditions were maintained throughout the range of tested airflow rates without descending into anoxic conditions. Therefore, from Fig. 3, it can also be concluded that the IS level is the more significant operational parameter. In addition, Fig. 4 graph confirms the model’s robustness. It compares and correlates the actual experimental results of the response parameter crude EPS with the ones predicted by the model, with an *R*^2^ value of 0.979 that proves no significant difference between the model prediction and the reality.

**Fig. 5:**
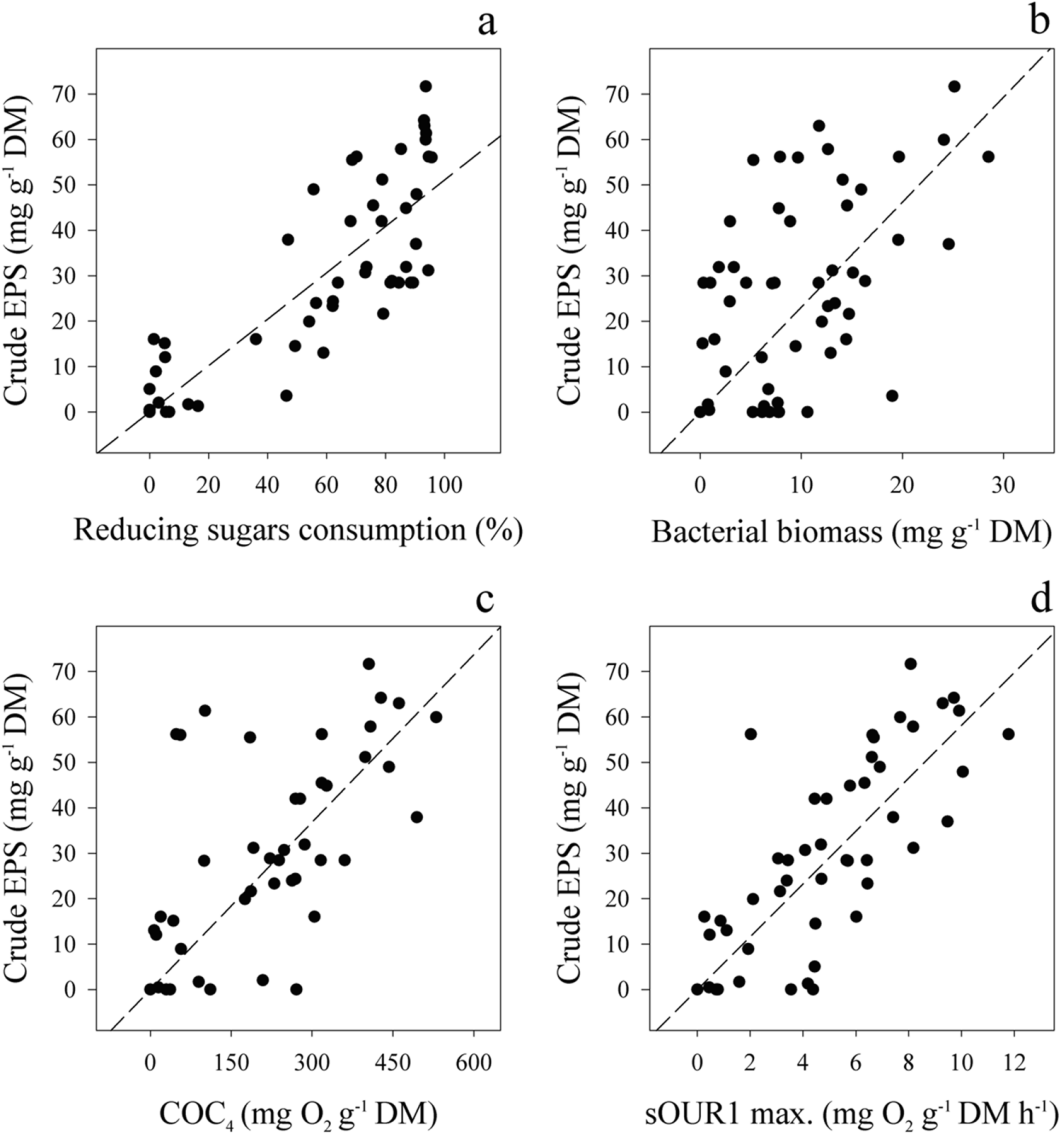
Correlation between crude EPS production and monitoring parameters from screening and optimisation experiments. **a** Reducing sugars consumption. **b** Bacterial biomass growth. **c** Cumulative oxygen consumption in 4 days (COC_4_). **d** Maximum specific oxygen uptake rate (sOUR max)

Complementarily to these results, it has been shown in previous studies that an increase in the consumption of some available sugars from substrates was related to EPS production (Joulak et al. 2022). In addition, Turakhia and Characklis (1989) suggested that EPS production by *Pseudomonas* or *Burkholderia* strains can be a linear function of bacterial growth rate. At the same time, the respiration indices COC_4_ and sOUR are directly related to the microbial growth rate in a fermentation system (Ponsá et al. 2010). Thus, from all the experimental results, including the time course and the optimisation steps of GJW and BJW combined with *B. cepacia*, some regression plots were generated and are shown in Fig. 5, comparing EPS production with other analysed response variables. Figure 5a shows the correlation of crude EPS yield with the consumption of reducing sugars expressed in percentage regarding the initial level of reducing sugars (*y* = 0.511*x*; *R*^2^ = 0.892). In Fig. 5b, bacterial biomass and crude EPS production are correlated (*y* = 2.310*x*; *R*^2^ = 0.677). While Fig. 5c presents the correlation of EPS yield with the cumulative oxygen consumption in the four (4) more productive days (COC_4_) (*y* = 0.123*x*; *R*^2^ = 0.784), Fig. 5d displays the correlation with the specific oxygen uptake rate as an average of the one hour of maximum activity (sOUR1 max.) (*y* = 5.816*x*; *R*^2^ = 0.867). The common trend in all the cases is that EPS yield increases when it augments the bacterial biomass and its consequent parameters, sugars, and oxygen consumption. These correlations suggest that SSF conditions affected several system response parameters similarly. Consequently, EPS production, which is related to all these factors, is expected to behave according to them.

## Conclusions

This study presents an attractive approach to the value chain of the agri-food industry by introducing a proof of concept that suggests alternative valorisation pathways for agri-food solid waste management using SSF to obtain added-value EPS biostimulants. Specifically, after a conscious screening, it was established that the combination of *B. cepacia* with GJW was the most appropriate of the assessed to perform a complete study of the process dynamics and to optimise three operational variables (specific airflow rate, inoculum size, and micronutrients concentration) to maximise EPS production. After optimisation, the best-found conditions were an airflow rate of 0.05 (± 0.01) L h^−1^ g^−1^ DM, an inoculum of 8.36 (± 0.97) E + 09 CFU g^−1^ DM, and a micronutrients concentration of 0.07 (± 0.01) mL g^−1^ DM to produce up to 71.1 (± 3.2) mg crude EPS g^−1^ DM.

Results also show that EPS production in the proposed system correlates with some monitoring parameters, such as sOUR, biomass, and reducing sugar consumption, indicating potential ways to exploit the advantages of the SSF system in further scaled scenarios. Although the results are valid for the specific combination, this study could serve to further explore the feasibility of using other similar agroindustrial streams, and it contains sufficient details to address the scale of the system.

## Data Availability

The data that support the findings of this study are available from the corresponding author upon reasonable request.
